# Unexplained First Trimester Intrahepatic Cholestasis of Pregnancy: A Case Report and Literature Review

**DOI:** 10.1155/2019/4980610

**Published:** 2019-12-27

**Authors:** Anastasia A. Salame, Mohammad J. Jaffal, Marco A. Mouanness, Amal R. Nasser Eddin, Labib M. Ghulmiyyah

**Affiliations:** American University of Beirut Medical Center, Beirut PO Box: 11-0236, Lebanon

## Abstract

Intrahepatic cholestasis of pregnancy (ICP) is a condition that usually affects the 3^rd^ trimester-pregnant women and is associated with adverse pregnancy outcomes. We present a 31-year-old G2P1 patient with symptoms of ICP as early as 10 weeks of gestation (WG). Her pruritis was initially attributed to eczema. Due to the intensity of her discomfort and failure of topical treatment, ICP was suspected, total bile acid salt levels were taken and ursodeoxycholic acid was subsequently started at 18 WG. The patient was followed closely during her pregnancy to adjust the dose of the medication accordingly. Induction of labor was performed at 37 WG without complications. This case demonstrated the importance of clinical suspicion in the setting of such symptomatology in order not to miss or delay treatment of threatening conditions such as ICP.

## 1. Introduction

Intrahepatic cholestasis of pregnancy (ICP) is a suspected endocrino-metabolic disease that affects pregnant women usually during the third trimester of pregnancy when the sex steroids reach their highest levels. While its maternal repercussions are somehow limited to minor symptoms of pruritis and related discomfort, pregnancy outcomes might be severely affected. Preterm labor and delivery, fetal hypoxia, and intra-uterine fetal demise (IUFD) are well known complications of ICP that has driven guidelines to recommend induction of labor during the early third trimester period [1]. The overall estimated rate of adverse pregnancy events ranges between 19.2% and 64.1% [2]. The exact pathophysiology of ICP leading to the accumulation of bile acid, the increasing serum liver enzymes, and the unfavorable pregnancy outcomes, remains unknown [2, 3].

Suggested risk factors for ICP include elevated estrogen levels, genetic causes, ethnicity, anticardiolipin antibodies, hepatitis C infection, multiple pregnancies, pregnancies resulting from assisted reproductive technologies as well as a personal history ICP [2, 4–6].

The diagnosis of ICP is made by the documentation of elevated bile acid serum levels (≥10 micro-mol/L). Depending on the serum bile acid levels, ICP can be classified as either mild (10 and 40 micro-mol/L, with the usual symptom of itching) or severe (>40 micro-mol/L, or concomitant gestational hypertension, IUFD and/or recurrent ICP) [7, 8].

ICP is considered as a disease of the third and/or late second trimester; however, recent evidence suggests that in rare situations ICP can be diagnosed as early as the first trimester. Numerous studies have suggested a link between the gestational age at which ICP was diagnosed and the pregnancy outcomes (increased risks of preterm delivery, small for gestational age, and stillbirth with earlier onset types of ICP) [9, 10]. We present a case of severe ICP in a spontaneous first trimester pregnancy with a successful outcome.

## 2. Case Report

A 31-year-old G2P1A0L1 presented to our clinic for follow up at 20 weeks of gestation with the diagnosis of ICP. The patient reported that her symptoms started as early as 10 weeks of gestation of her spontaneously conceived pregnancy. Initially the patient used topical steroids in an attempt to relieve her symptoms to no avail. She later sought medical attention from several dermatologists who prescribed creams for eczema and dermatitis that included lotions, antihistamines and oral steroids reaching 40 mg daily also to no avail. Despite two weeks of treatment the pruritis increased in severity and at this point ICP was suspected for which total bile acid salts TBAS as well as liver function tests were ordered. The patient discontinued her previous medications and was started prophylactically on ursodeoxycholic acid (UDCA) at a dose of 250 mg TID. At 18 weeks of gestation the results of her laboratory tests showed an increase in TBAS of 9.1. At this point the patient was referred to a maternal fetal medicine specialist at our center.

**Figure 1:**
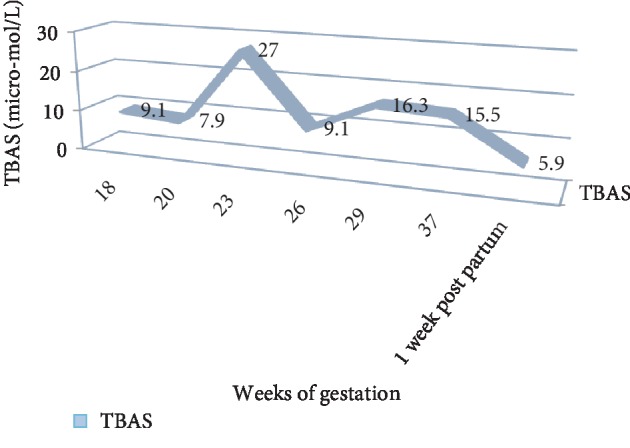
TBAS values in micro-mol/L as a function of weeks of gestation.

At our clinic, the patient was followed every 3 weeks with out-patient clinic visits until 33 weeks of gestation and then weekly afterwards. Nonstress tests were performed twice weekly as of 28 WG and follow up growth scans at 2-3 week intervals. The estimated fetal weight was within 50th percentile range at all times. The levels of TBAS were measured every 3 weeks. Her UDCA doses were modified depending on her serum bile salt values. The patients' TBAS values are presented in Figure 1.

By the end of her pregnancy she was receiving 2000 mg daily of UDCA with mild tolerable symptoms. Her liver function tests were within the normal limits at all times during pregnancy. SGOT values ranged between 13 and 20 U/L, SGPT values ranged between 8 and 14 U/L, while bilirubin was less than 0.1 mg/dl.

The patient was induced at 37+1 weeks of gestation using misoprostol and then followed by oxytocin for labor augmentation. The patient progressed smoothly to full dilation within 12 hours of induction. She delivered a live born male of weight 3340 grams and Apagr score of 8 and 9 at 1 and 5 minutes respectively. The patient and her child were discharged home on the day 1 post vaginal delivery in a stable condition. Follow up TBAS 1-week post-partum revealed a value of 5.9 micro-mol/L, with complete resolution of symptoms. The patient provided an informed consent with which we were given permission to access her medical chart.

## 3. Discussion

The prevalence of ICP during pregnancy varies geographically from 0.1 to 15.6% [11]. The potential poor pregnancy outcomes push obstetricians towards a closer follow up of affected pregnancies. One of the challenges that primary care physicians face is the ability to make a timely diagnosis of such a threatening condition, especially when symptoms appear early on during pregnancy. The cut-off gestational age for differentiation between early and late onset is set at 34 weeks of gestation [5]. It is believed that elevated levels of estrogen as well as progesterone might play an inducing factor in the onset of ICP [12, 13]. Add to that, genetic predisposition might lead to malfunction in the bile acid transmembrane hepatocyte transporters, causing accumulation of bile acid. In fact, in the setting of hyperestrogenism (i.e. pregnancy or oral contraceptive pill intake), this malfunction becomes evident and the patient develops the typical symptomatology [14]. Most of the data documented that early onset first trimester ICP cases are concomitant with supra-physiological estrogen levels during the first trimester. Such elevated sex steroid levels are usually noted in multiple gestations or pregnancies complicated by ovarian hyperstimulation syndrome. Add to that, pregnancies conceived via ovarian stimulation cycles with subsequent embryo transfer are at a higher risk of developing early onset ICP [15]. However, our patient conceived spontaneously and already had a noncomplicated pregnancy before which only strengthens the fact that the cause-effect relationship leading to ICP is weak and still needs to be fully clarified.  Table 1 shows the published cases of first trimester ICP along with the identified risk factors and the pregnancy outcomes.

J. Lin et al. showed that adverse pregnancy outcomes were higher in patients diagnosed to have early onset ICP compared to the late-onset ICP group, the analysis revealed significantly higher rates of fetal distress, intrauterine growth restriction, preterm birth, and low birth weight in the early-onset ICP group [5]. The overall preterm delivery rate was 34.15% in comparison to the worldwide numbers that range between 19 and 60% [5, 9, 16]. This is due in part to the induction of labor at earlier gestational ages to avoid fetal adverse events. Add to that, elevated bile acid concentrations alter cell membrane permeability and stimulate prostaglandin release, thus leading to increased uterine reactivity as well as increased sensitivity to oxytocin which might cause preterm labor [17]. On another level, it has been postulated that bile acid salts deposit in the placenta and lead to edema, strictures, and acceleration of placental cell apoptosis with subsequent alteration of the placental function [9]. Also, elevated maternal and amniotic fluid levels of bile acids can lead to umbilical vessels spasms with resultant reduction in the trans-placental nutrient exchange and fetal oxygenation. It has been postulated as well that, elevated fetal levels of acids might cause end organ damage resulting in fetal hypoxia and death [18]. Finally, recent studies have showed that fetal cardiomyocytes harbor receptors for bile acids known as the nuclear BA receptor farnesoid-X receptor (FXR) that have been found to be involved in cardiac injury and cardiomyocyte apoptosis [19]. Elevated levels of bile acids might therefore be potentially cardio-toxic with resultant life threatening arrhythmias, cardiac dysfunction, and intrauterine fetal death (relative risk of 2.58 in comparison with non-ICP pregnancies) [20]. In the setting of early onset ICP, the duration of fetal and placental exposure to abnormally elevated bile acid salt levels is significantly prolonged and thus the cytotoxic effects are more likely to take place. This mandates a closer follow up of patients diagnosed to have ICP at an early stage of pregnancy as well as timed intervention to interrupt pregnancy [9, 18].

Until today, many studies have been performed to assess the effectiveness and safety profile of the widely used Ursodeoxycholic acid (UDCA). It has been suggested that it not only improves the severity of the symptoms related to ICP, but also decreases the level of bile acid salts and potentially improves the pregnancy outcome especially in the setting of early onset ICP [21, 22]. The exact mechanism by which UDCA improves the outcome of ICP patients is unknown so far. It is believed that UDCA normalizes hepatic metabolism, improves transplacental bile acid transport, and/or prevents the oxidative stress and cellular death [23]. Furthermore, UDCA is believed to be cardio-protective and thus an enhancer of the fetal cardiac function. This is vital in the setting of the suspicion that elevated bile acid salts can cause cardiac arrest of the fetus [24]. In Lebanon, clear and independent guidelines are absent, thus the guidelines of the American college of obstetrics and Gynecology (ACOG) as well as the Society for Maternal-Fetal Medicine (SMFM) are adapted in our daily practice. However, given that this particular patient did not improve on the recommended doses of UDCA, the decision was taken to treat her with the highest level recommended in the literature which is equivalent to 2000 mg/day [8]. Other newer approaches to ICP medical treatment such as Resveratrol, a Silent Information Regulator 1 (SIRT1) is thought to activate SIRT1 with resultant reduction in the levels of pro-inflammatory cytokines and bile acids [25]. The use of Resveratrol in the context of ICP however is still limited pending more conclusive data.

The ideal time of delivery is determined based on the severity of maternal symptoms, onset of ICP, and maternal discomfort as well as assessment of fetal well-being [5]. Mild ICP patients can be managed till 38 WG, while severe cases of ICP should be delivered at maximum of 37 WG. Recent investigations have showed that ICP patients are more prone to serious conditions such as preeclampsia, gestational diabetes, as well as nonpregnancy related hepatobiliary disease such portal hypertension and liver failure [26–28].

## 4. Conclusion

First trimester ICP is a rare condition that physicians need to be aware of particularly in the setting of typical symptomatology. So far, close follow-up, fetal monitoring, and UDCA are the mainstay of treatment to avoid poor neonatal outcomes. Further studies are needed to better understand the pathophysiology and find the ultimate treatment for ICP.

## Figures and Tables

**Table 1:** Characteristics of all cases of first trimester ICP documented in the literature.

Article title	Number of cases	Predisposing factor	Ethnicity	Gestational age at diagnosis/onset of symptoms	Bile acid level at the time of diagnosis (micro-mole/L)	Pregnancy Outcome
Kirkinen et al. 1995	1	(i) Triploid fetus	Not available	Not available	Not available	Not specified
		(ii) Elevated alpha Fetoprotein				

Brites et al. 1998	1	Not specified	Not available	11 weeks of gestation	198 at 20 weeks of gestation	IOL at 36 weeks of gestation.
						Baby had intestinal stenosis

Zamah et al. 2008	2	(i) IVF treatment	Asian	8 weeks of gestation	137.2	IOL at term due to oligohydramnios
		(ii) Moderate OHSS	East Indian	6 weeks of gestation	308	Spontaneous miscarriage at 9 weeks of gestation

Steele et al. 2012	1	(i) First pregnancy No obvious risk factors	British	11 weeks of gestation	205 at 16 weeks of gestation	Emergency cesarean for nonreassuring fetal heart at 32^+2^ weeks of gestation

Johnston et al. 2014	1	(i) Recurrent ICP	Not available	8 weeks of gestation	217	PPROM at 30^+5^ weeks of gestation
		(ii) Heterozygous mutation of ABCB4 gene				c/s for malpresentation

Hubschmann et al. 2016	1	(i) Recurrent ICP	Guatemalan	7^+5^ weeks of gestation	243.6 at 13^+5^ weeks of gestation	Elective cesarean section at 31^+6^ weeks of gestation

Mutlu et al. 2017	2	(i) IVF treatment	Turkish	6 weeks of gestation	166.8	Blighted ovum
		(ii) Moderate OHSS	Turkish	5 weeks of gestation	122	PPROM at 36 WG with delivery of twins via cesarean section

Our case	1	(i) No known risk factors	Lebanese	10 weeks of gestation	9.1 at 18 weeks of gestation	IOL at 37 weeks of gestation.
